# Virtual Reality Gait Training to Promote Balance and Gait Among Older People: A Randomized Clinical Trial

**DOI:** 10.3390/geriatrics6010001

**Published:** 2020-12-22

**Authors:** Kyeongjin Lee

**Affiliations:** Department of Physical Therapy, College of Health Science, Kyungdong University, Gosung 24764, Korea; kjlee@kduniv.ac.kr

**Keywords:** elderly, virtual reality, postural balance, gait

## Abstract

Falls are the leading cause of injury and injury-related death in the elderly. This study evaluated the effect of virtual reality gait training (VRGT) with non-motorized treadmill on balance and gait ability of elderly individuals who had experienced a fall. Fifty-six elderly individuals living in local communities participated in this study. Subjects who met the selection criteria were randomly divided into a VRGT group (*n* = 28) and a control group (*n* = 28). The VRGT group received VRGT with non-motorized treadmill for 50 min a day for 4 weeks and 5 days a week. The control group received non-motorized treadmill gait training without virtual reality for the same amount of time as the VRGT group. Before and after the training, the one-leg-standing test, Berg Balance Scale, Functional Reach test, and Timed Up and Go test were used to assess balance ability, and the gait analyzer system was used to evaluate the improvement in gait spatiotemporal parameters. In the VRGT group, the balance ability variable showed a significant decrease in the one-leg-standing test and a significant improvement in the Timed Up and Go test. With respect to spatiotemporal gait parameters, velocity and step width decreased significantly in the VRGT group (*p* < 0.05), and stride length and step length were significantly improved in the VRGT group (*p* < 0.05). VRGT with non-motorized treadmill has been shown to improve balance and gait ability in the elderly. This study is expected to provide basic data on exercise programs for the elderly to prevent falls.

## 1. Introduction

Gait is a complex motion that occurs when the nerves, musculoskeletal, and sensory systems are collectively involved, and various segments and joints of the body are harmoniously coordinated. Movement and gait disorders are the first problems that appear in the elderly because of the decrease in muscle strength and balance ability along with the aging process [1,2]. The decline in physical functioning of older people often leads to instability in walking and a decrease in balance ability, thereby limiting social autonomy of older adults and causing falls [3]. Since most falls occur while walking, impaired walking is associated with an increased risk of falls [4,5]. As the older population is expected to increase to 88 million by 2050, these falls will become a more serious and prevalent problem [6].

Recently, due to the influence of the modern aging society, various studies on walking and posture control have been conducted on posture, balance, and stability of the elderly. Compared to young adults, the general older population’s gait pattern has a shorter step time and they spend a longer time in double support stance [7]. These features need to be considered to make walking more secure and stable for the elderly [8,9]. In addition, in order to maintain balance and stability during walking, the elderly have decreased cadence and stride length, increased support base, and decreased walking speed [7,8]. These older individuals suffer a fundamental cause of instability when walking, and in order to solve the cause, an exercise method that can improve and stabilize the gait of older adults is needed.

Treadmill training is widely used for gait training. The treadmill is an excellent alternative to ground training because it is easy to control the walking conditions, such as speed and slope, and frees the individual from time and space constraints [10]. Treadmill walking is generally similar to walking on flat ground [10,11], but a relatively narrow and straight walking path is used in a treadmill, and there is a difference between the speed fixed by the belt and the constant surface of the belt, visual flow, and vestibular perceptual information [12,13]. Compared to electric motorized treadmills, non-motorized treadmills have curved surfaces without a motor, so the runner has to stride at the surface and propel the belt with each stride. The non-motorized treadmill is recommended for older people with weak knees as it effectively reduces the impact on the knee due to small vertical deformation. Previous studies have used motorized treadmills for gait analysis or training [14,15], but studies using non-motorized treadmills in gait rehabilitation have been insufficient in number.

For gait training in the elderly, not only intensive training, but also experience in various environments and changing situations when walking is required [14,16]. Cognition and obstacle negotiation abilities as well as physical ability are important for safe walking. Virtual reality has emerged as a potential technology that promotes physical activity and improves both the motor and cognitive aspects of walking [17,18]. Virtual reality refers to an advanced computer interface that includes real-time simulation and interaction through multiple sensory channels. It has been found that the sensory information provided by VR is similar to the sensation that can be felt in a real environment [14,16]. Virtual reality training can provide education in a stimulating and rich environment aimed at improving motor and cognitive functions. It also provides feedback on performance to help learn new exercise strategies in a variety of risk-controlled environments. Various studies have been conducted using a virtual reality-based treadmill gait training for Parkinson’s disease, patients with stroke, and the elderly [14,15,19]. It was found that virtual reality-based training was more effective in improving the motor function of patients than existing task-oriented interventions and had a positive effect on active training participation and motivation. Virtual reality-based treadmill training effectively improves balance and gait, but has a disadvantage of causing nausea or vomiting in the subject due to the discrepancy between the image and the walking speed [20]. The speed of visual flow on a moving screen is an important factor in controlling posture when walking. Matching the visual flow according to the walking speed as in real life, where the virtual reality image is not at a constant reproduction speed, is more effective in improving walking function [21]. Virtual reality training was effective in walking training for stroke, cerebral palsy and amputation patients, as it could promote motor learning by providing effective intrinsic and external feedback [22,23,24]. In particular, since visual feedback plays a vital role in gait training, it was confirmed that gait training through real-time feedback based on virtual reality effectively improves gait performance [24,25].

In recent years, smartphones have become a necessity in everyday life and are devices that the elderly use a lot. It will be exciting and useful to use these smartphone applications to replace complicated and expensive virtual reality equipment.

Therefore, in this study, we propose an excellent intervention in motion-tracking technology that can adjust the video speed in real-time according to the movement of walking using a smartphone and propose a method of applying gait training using non-motorized treadmill for the elderly. In addition, in order to prevent falls, this study intends to confirm the effect on balance and gait by applying virtual reality gait training (VRGT) to the elderly living in local communities.

## 2. Materials and Methods

### 2.1. Subjects

The subjects of this study were senior citizens residing in local communities and were recruited through advertisements at senior welfare centers. The inclusion criteria were: (1) aged 65 years or above who had experienced more than one fall in the past year, (2) able to walk for 8 min unassisted; and (3) had a Mini-mental state examination score over of 24 or higher, with no cognitive problems. The exclusion criteria included: (1) dementia; (2) had history of stroke, traumatic brain injury or other neurological disorders; (3) the presence of orthopedic disease interfering with participation in the study; and (4) unable to comply with the training or currently participating in another interfering therapy or a fall clinics program. All subjects voluntarily signed consent to participate in the study and the study was approved by the institutional review committee of Kyungdong University (1041455-201706-IR-012-01).

### 2.2. Determination of the Sample Size

This study used a single blind design for Randomized Controlled trials. G-Power 3.19 software (Heinrich Heine University Düsseldor, Düsseldorf, Germany) was used to calculate the sample size, and the alpha error probability and power were set to 0.05 and 0.8, respectively. The effect size was set to 0.8, according to Cohen’s method. More than 26 samples per group were required, and 28 people were assigned to each group, considering a 10% dropout rate.

### 2.3. Procedure

**Figure 1:**
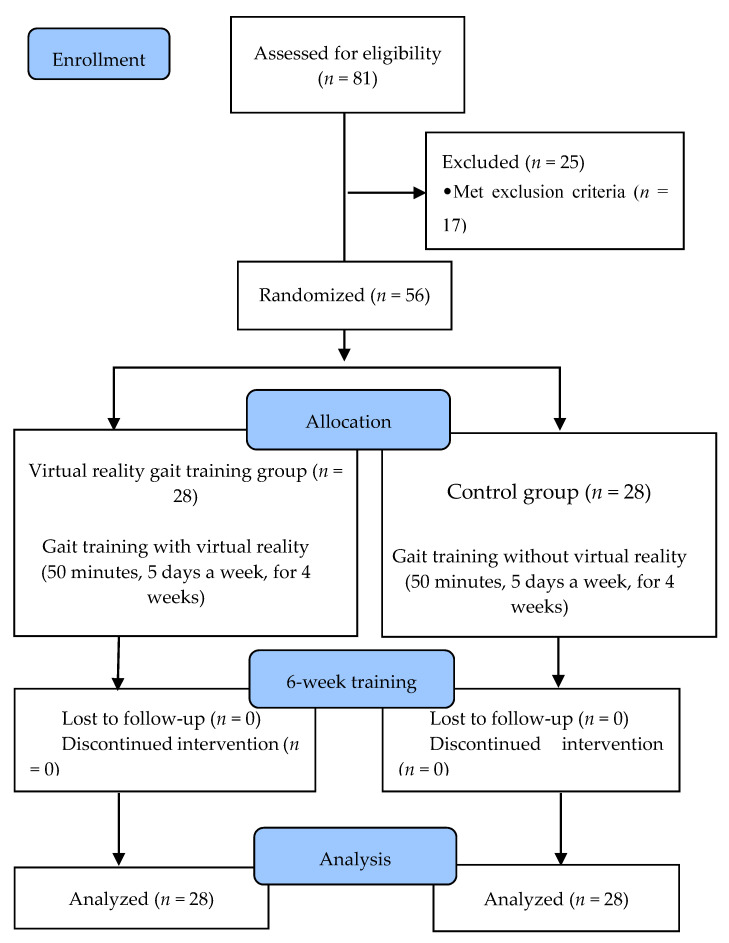
Flow diagram of the study.

Fifty-six subjects who met the inclusion criteria were divided into a VRGT group (*n* = 28) and a control group (*n* = 28), using random allocation software (version 1.0) to minimize selection bias. There were no significant differences in sex, age, height, weight, and fall experience between the two groups. The individuals in VRGT group performed VRGT 5 times a week for 4 weeks and 50 min per session, while the control group performed standard treadmill training without virtual reality 5 times a week for 4 weeks and 50 min per session. The subjects’ general characteristics, postural balance, and walking ability were evaluated by two blind subjects who were not aware of the subjects’ group. The exercise program was implemented at the M Senior Welfare Center in Gyeonggi Province, South Korea and was safely performed by an experienced physical therapist. Accidents were prevented with the help of one experimental assistant. Before the exercise program, all subjects were informed about the exercise and given education on the prevention of falls in the elderly for an hour. Training consisted of 10 min of warm-up exercise (5 min stretching, 5 min slow walking), 30 min of main exercise, and 10 min of cool-down exercise, which was a total of 50 min. All participants showed a participation rate of over 80%, and no one dropped out (Figure 1).

### 2.4. Virtual Reality Gait Training with Non-Motorized Treadmill

The warm-up consisted of 5 min of stretching and 5 min of walking. Since a too sudden start of walking on the treadmill may result in dizziness, the subject was allowed to walk for 3 min at a speed slightly slower than the normal speed of the subject, and then walk for 2 min at the usual speed. Electric motorized treadmills, which are often used for gait training, have a flat running surface with a motor that propels a continuous moving belt in which the runner tries to match the speed through stride and stride length. On the other hand, a non-motorized treadmill has a curved surface without a motor, so the runner must stride on the surface and propel the belt with each stride. The non-motorized treadmill is most similar to ground movement in that the belt does not use power, so the user must actively pull it with his leg [26,27]. In addition, the non-motorized treadmill is effective in reducing the impact on the knee because of its small vertical deformation [28]; therefore, it is recommended for older individuals with weak knees. In this study, a non-powered treadmill (Jubatus, Irunner, Seoul, South Korea) was used to induce active participation of the subjects. Before walking training in virtual reality, the subject was trained to adapt sufficiently for safe training. When dizziness occurred during the flow of the video, a break was taken. The subject was instructed to start walking more slowly than usual and walk at the usual speed. Subjects walked while staring at the curved 65-inch screen TV (UN65MU6500FXKR, Samsung, Seoul, South Korea) in front of the treadmill. For the safety of the subject, training was conducted under the supervision of two therapists. Subjects wore shoes but did not use a walker. During this exercise, the videos used in the VRGT group were approximately of 8-min duration, and subjects were provided with three such videos. Each subject took a 3 min break during training as and when needed. The rest time was adjusted according to the subject’s condition. The main exercise in the control group was performed in the same way as the VRGT group without video. The therapist continuously monitored the subject and asked the subject to stop training if fatigue or difficulty in walking was detected due to breathing or a change in facial color, before resuming training after taking sufficient rest. Subjects were instructed to stop at will at any time if there were any abnormal symptoms. After this exercise, the subjects were allowed to walk at a slow speed for approximately 5 min. The subjects got down from the treadmill, took enough rest, and were observed until their breathing and pulse rate recovered. Both groups received health training for fall prevention. Educational topics included fall causes, fall risk factors, fall prevention exercises, surroundings, and facilities.

### 2.5. Virtual Reality Device and Motion Tracking

A smartphone running application (Virtual Active, BitGym, Los Angeles, CA, USA) was used to provide virtual reality images according to the walking speed. This application provides images of the tourist attractions across the world, such as valleys, mountains, beaches, and city streets, captured by experts. For this study, we included images of the Harbor Bridge and Opera House in Sydney, Australia; Grand Canyon and Chicago River Walk in the United States; the riverbanks in Venice, Italy; and downtown Las Vegas, United States. The subjects were very interested in the scenarios and actively participated in the study.

If the screen’s image speed and the subject’s walking speed do not match, it is difficult for the subject to immerse in the image on the screen. On these occasions, the screen image is not virtual reality, and the image on the screen is just a typical landscape image. Therefore, in this study, the subject’s movement and the screen’s optical flow were synchronized. The faster the participant walks, the faster the screen speed, and the slower the participant walks, the slower the screen. The flow of the provided image and the subject’s movement were synchronized to immerse in the virtual reality situation. During gait training, the subject will experience virtual reality as if entering the environment on the screen. We set up the smartphone camera facing the subject. When the subject starts walking, the smartphone recognizes three virtual landmarks on the head and each shoulder and draws a movement pattern. There is no other movement nearby, so the camera can easily recognize the body’s movement while walking. The human body has a repetitive pattern of movement while walking. The smartphone application creates a pattern by recognizing repeated movements for 5 s and controls the screen’s video speed according to the pattern.

The video from the smartphone was mirrored on a curved 65-inch screen TV placed in front of the treadmill. Six treadmills were used to exercise 56 subjects, and screens and treadmills were installed in three rooms to give virtual reality gait training to the subjects in the VRGT group.

### 2.6. Outcome Measurements

To measure postural balance, the one-leg-standing (OLS) test, Berg Balance Scale (BBS), Functional Reach test (FRT), and Timed Up and Go (TUG) test were used. The OLS measures the length of time the subject stands with arms crossed on the non-dominant leg. Time is measured before the other foot touches the floor. The measuring tool was a stopwatch and recorded in seconds. The BBS is used to assess the risk of falls by assessing the dynamic balance of the elderly or patients at risk of falls [29]. Each item is evaluated on a 5-point scale and consists of 14 items necessary for daily life. The higher the score, the better the balance and lower the risk of falling. The FRT evaluates the limits of stability and indicates the degree of dynamic balance and flexibility [30], along with the distance the subject can extend an arm to the fullest level while standing horizontally. The TUG tests dynamic balancing ability and mobility. The results are used to assess the risk of falls [31]. The subject rises from a 46 cm high armrest chair, walks a distance of 3 m, returns and measures the time to sit back on the chair. This sequence is measured in seconds using a stopwatch. All tests except BBS were performed 3 times, averaged, and recorded.

A gait analyzer (OptoGait, Microgate S.r.l, Bolzano, Italy) was used to evaluate gait ability. Gait ability was expressed as a spatiotemporal parameter. The gait analyzer consisted of two 1-m long transmit and receive bars and a webcam. The bars on both sides consist of a receiving bar and a transmitting bar and are placed 3 m apart on a flat floor. Each bar is equipped with light-emitting diodes at 1 cm intervals, and the two bars communicate through continuous infrared transmission from the transmission bar to the receiving bar. During walking, the subject’s foot is detected between the communication transmission and reception bars and walking parameter data are collected. The video information stored on the webcam was used to accurately synchronize the measured gait. After receiving a verbal signal from the tester, the subject walked at a normal speed and recorded data of 3 m in the middle of a total of 5 m. The collected gait parameter data were processed with OptoGait software (Version 1.11, Microgate, Bolzano, Italy). For the accuracy of data collection, pretest zero calibration was performed, and tests were performed without walking tools, such as walking aids or weight-bearing suspension devices. The average of the values recorded by repeating measurements three times was used as the measured value.

### 2.7. Statistical Analysis

Descriptive statistics were used to summarize the subjects’ baseline characteristics. The Shapiro-Wilk test was used to check the normality of all variables. To investigate the differences between groups at baseline, we performed an independent *t*-test for continuous variables and a chi-square test for categorical variables. Comparisons between pretreatment and posttreatment data within each group were analyzed using a paired *t*-test. An independent *t*-test was used to evaluate the change in the mean score of the variables between the VRGT group and the control group at baseline and 4 months after intervention. All analyses were performed using SPSS version 19.0 (IBM Corporation, Armonk, NY, USA) for Windows, and a *p* value < 0.05 was considered statistically significant.

## 3. Results

In the VRGT group and the control group, there was no statistically significant difference in all data of the two groups at baseline. Fifty-six subjects from both groups participated in the training, and all of them performed statistical analysis without dropouts (Table 1).

Table 2 shows the changes in postural balance including OLS, BBS, FRT, and TUG before and after intervention. The changes in OLS and TUG showed a significant improvement after intervention in the VRGT group (*p* < 0.05), but not in the control group. The BBS and FRT were not significantly changed after intervention in both groups. Outcome measures of temporal gait parameter and spatial gait parameter of the VRGT and control groups are shown in Table 3 and Table 4. The changes in velocity and step width variables showed after intervention in the VRGT group (*p* < 0.05), but not in the control group. The changes in static stride length and step length variables showed significant improvement after intervention in both groups (*p* < 0.05). However, the VRGT group showed significantly more improvement as compared to the control group (*p* < 0.05).

## 4. Discussion

Human posture control relies on the integration of information input from various sensory stimuli and, in particular, the vision plays an important role in dynamic stability control. Virtual reality enables humans to perceive and immerse their brain in the visual world closest to the real three-dimensional world [32]. It is a great advantage to provide virtual reality to the elderly who have limited daily activities [14,17,18].

In this study, VRGT, which uses motion tracking technology that synchronizes gait speed and virtual reality optical flow, was utilized by elderly individuals and its effects on balance and gait were investigated. Balance is an important indicator for predicting falls, and many studies have evaluated BBS, FRT, and OLS [29,30,31]. In this study, OLS showed a significant improvement of 30.6% and TUG showed a significant decrease of 10%, confirming that VRGT is effective in improving postural balance. OLS, although simple, has been used as a useful tool for assessing the risk of falls [33]. Studies of other interventions to prevent falls in the elderly have shown a wide range of results from 3 to 40 s when standing with eyes open [34]. Previous studies have reported the time of OLS according to age [35]. In particular, in the case of individuals in their 80s, the range of OLS results is so wide that direct comparison between studies is difficult; however, the improvement in OLS time in this study is thought to be a result of the improvement of lower limb muscle strength through gait training. While using the non-motorized treadmill, an individual must walk on a curved belt without a motor, which requires greater involvement of lower extremity strength than a motorized treadmill that carries the leg through each stride at a constant belt speed [26,27]. The OLS motion accounts for 40% of the gait motion and enables the swing of the opposite leg. Therefore, the OLS test is closely related to mobility and balance ability, including gait [33,36]. The practical cutoff value recommended for TUGs indicating normal and subnormal performance was 12 s [37]. The Bischoff et al. [37] study concluded that the TUG time was 6.0 to 11.2 s for women aged 65 to 85 years living in local communities, and that they should be able to perform the TUG test within 12 s. In this study, the TUG time was significantly reduced from 13.24 s to 11.92 s in the experimental group, which lowered the risk of subjects with high risk of falls. Through the results of the indicators related to postural balance, VRGT was demonstrated to be effective in improving balance, an important indicator of fall risk.

Due to a decrease in gait ability, the older individual develops a gait pattern to increase safety, while their ability to move the body forward decreases [8,9]. Older people try to increase stability by increasing the time spent in the double support stance while reducing gait speed and making the step length shorter [8]. Gait evaluation is used as a guideline for falls, and some studies have suggested that, among the spatiotemporal variables of walking, changes in velocity and step length increase the risk of falls [38,39]. As a result of the training in this study, velocity increased by 2.9%, step length and stride length showed a significant improvement of 4.2% and 4.1%, respectively, and step width showed a significant decrease of 8% in the studied population. Previous studies also proved that virtual reality training has a positive effect on the improvement of the older individual’s gait ability, and in particular, the combination of virtual reality and treadmill training was found to be effective in improving gait ability [40]. In the Mirelman et al. [40] study, walking speed was improved by 8% with non-immersive virtual reality treadmill training, which is similar to the results shown in this study. Considering that gait speed is a major factor in predicting community mobility, VRGT can be an effective approach to improve the gait ability of the older population. In a previous study that examined the relationship between the spatiotemporal variables of gait and falls in 890 people aged 65 to 90 years old, among many variables, stride length was found to be the most commonly correlated with falls [41]. Liao et al. [18] reported a significant improvement of 10% in stride length by performing virtual reality-based physical and cognitive training in older patients. This improvement in gait ability activates the cortex through task-oriented training, which shows the importance of feedback stimulation [15,18,19,20,40]. In this study, it is thought that the improvement of spatio-temporal walking ability was induced by providing multisensory feedback stimulation for various environments during VRGT. Virtual reality provides many opportunities to train the visual attention in a safe and controlled environment; therefore, it is believed that the sense of immersion has led to an increased recognition and focus on walking movements. In addition, in this study, it is believed that the visual information of the image and the gait speed were matched, and gait ability improved by the integration of vision and the intrinsic sense. When gait speed is increased, the visual flow of the screen is accelerated, and the speed is increased, therefore it was possible to induce interest and motivation or increase participation of the subjects in their training.

In general treadmill walking, since gait training is performed in a fixed visual information environment, a discrepancy between vision and proprioception is experienced. In this study, it is thought that the improvement in walking ability was achieved through the integration of sight and intrinsic sense through the matching of walking speed and captured images. The development and application of gait training that combines technologies such as augmented reality and mixed reality should be established as a quantitative and standardized therapeutic intervention model. This study can be of significance in presenting a new environment for walking training for the older population at a point where virtual reality is being applied in various fields, such as medical care, education, and culture. This suggests that the expansion of virtual reality for health-related interventions in a safe and controlled environment may have potential therapeutic benefits that promote independence and quality of life for older people.

This study has several limitations. Participants were trained for six weeks to test the effectiveness. However, in this study, we did not follow up on how effective the results remained over time. There was no evaluation of participants’ motivation to participate. Individuals’ dizziness, which often occurs in VR training, has not been investigated. In future studies, it is necessary to study the effect’s persistence through a long-term follow-up study. We also think research is needed to evaluate the psychological factors and factors that hinder training in more detail.

## 5. Conclusions

This study confirmed that virtual reality gait training using optic flow video in a non-motorized treadmill is useful for preventing falls and improving balance and gait in the elderly and shows the effectiveness and validity of virtual reality gait training using motion-tracking technology.

## Figures and Tables

**Table 1:** General Characteristics of Subject.

Variables	VRGT Group (*n* = 28)	Control Group (*n* = 28)	χ^2^/*t*	*p*
Age (year)	81.01 ± 6.89	79.47 ± 6.15	0.879	0.383
Height (cm)	166.36 ± 6.42	164.21 ± 8.58	1.058	0.295
Weight (kg)	63.12 ± 6.53	61.52 ± 8.62	0.782	0.438
BMI (point)	22.81 ± 2.14	22.83 ± 2.76	0.024	0.981
MMSE-K	25.86 ± 1.38	25.64 ± 0.91	0.686	0.496
Gender (male/female)	16/12	15/13	0.788	0.072

Values are expressed as mean ± standard deviation. The independent *t*-test and Chi-squared test are used to compare the dependent variables between the two groups. VRGT, virtual reality gait training; BMI, body mass index; MMSE, mini-mental state examination.

**Table 2:** Changes of postural balance.

Variables		VRGT Group (*n* = 28)	Control Group (*n* = 28)	χ^2^/*t*	*p*
OLS (sec)	Pre	28.82 ± 6.32	27.76 ± 7.01	0.594	0.555
	Post	37.64 ± 9.29	29.45 ± 7.40		
	Change Score	8.82 ± 3.58	1.69 ± 4.87	6.240	0.000
	*t*	13.026	1.837		
	*p*	0.000	0.078		
BBS (point)	Pre	46.18 ± 3.86	44.89 ± 4.59	1.135	0.262
	Post	46.39 ± 4.34	45.25 ± 4.64		
	Change Score	0.21 ± 1.10	0.36 ± 1.13	0.479	0.634
	*t*	1.030	1.676		
	*p*	0.312	0.106		
FRT (cm)	Pre	25.19 ± 5.31	25.45 ± 4.26	0.202	0.841
	Post	23.80 ± 5.38	25.39 ± 3.58		
	Change Score	−1.39 ± 7.17	−0.06 ± 5.73	0.764	0.448
	*t*	1.024	0.057		
	*p*	0.315	0.955		
TUG (sec)	Pre	13.24 ± 5.91	12.55 ± 4.48	0.486	0.629
	Post	11.92 ± 5.42	12.33 ± 4.86		
	Change Score	−1.31 ± 1.51	−0.22 ± 0.83	3.339	0.002
	*t*	4.600	1.431		
	*p*	0.000	0.164		

Values are expressed as mean ± standard deviation. VRGT, virtual reality gait training; OLS, one leg standing test; BBS, Berg balance scale; FRT, functional reach test; TUG, timed up and go test.

**Table 3:** Changes of temporal gait parameter.

Variables		VRGT Group (*n* = 28)	Control Group (*n* = 28)	χ^2^/*t*	*p*
Velocity (m/s)	Pre	1.02 ± 0.04	1.06 ± 0.14	1.529	0.132
	Post	1.05 ± 0.04	1.08 ± 0.09		
	Change Score	0.03 ± 0.04	0.02 ± 0.13	0.245	0.807
	*t*	3.452	0.816		
	*p*	0.002	0.422		
Cadence (step/min)	Pre	113.29 ± 20.77	121.63 ± 23.86	1.395	0.169
	Post	109.98 ± 12.95	119.65 ± 23.47		
	Change Score	−3.32 ± 9.24	−1.98 ± 9.24	0.541	0.591
	*t*	1.900	1.137		
	*p*	0.068	0.266		
Stride time (sec)	Pre	1.09 ± 0.17	1.02 ± 0.19	1.366	0.178
	Post	1.11 ± 0.13	1.04 ± 0.18		
	Change Score	0.02 ± 0.06	0.01 ± 0.06	0.154	0.878
	*t*	1.423	1.176		
	*p*	0.166	0.250		
Step time (sec)	Pre	0.54 ± 0.09	0.51 ± 0.10	1.366	0.178
	Post	0.55 ± 0.06	0.52 ± 0.09		
	Change Score	0.01 ± 0.03	0.01 ± 0.03	0.154	0.878
	*t*	1.423	1.176		
	*p*	0.166	0.250		

Values are expressed as mean ± standard deviation. VRGT, virtual reality gait training.

**Table 4:** Changes of spatial gait parameter.

Variables		VRGT Group (*n* = 28)	Control Group (*n* = 28)	χ^2^/*t*	*p*
Stride length (cm)	Pre	110.99 ± 16.29	109.17 ± 13.13	0.462	0.646
	Post	115.61 ± 12.06	111.29 ± 15.02		
	Change Score	4.61 ± 5.01	2.12 ± 3.60	2.136	0.037
	*t*	4.875	3.134		
	*p*	0.000	0.004		
Step length (cm)	Pre	55.50 ± 8.14	54.58 ± 6.56	0.462	0.646
	Post	57.80 ± 6.03	55.65 ± 7.51		
	Change Score	2.31 ± 2.50	1.06 ± 1.80	2.136	0.037
	*t*	4.875	3.134		
	*p*	0.000	0.004		
Step width (cm)	Pre	9.95 ± 2.34	10.13 ± 1.84	0.327	0.745
	Post	9.15 ± 2.25	10.47 ± 2.11		
	Change Score	−0.80 ± 0.40	0.34 ± 2.52	2.364	0.022
	*t*	10.713	0.709		
	*p*	0.000	0.485		

Values are expressed as mean ± standard deviation. VRGT, virtual reality gait training.
